# Ethyl 2-(3-phenyl­thio­ureido)-5,6-di­hydro-4*H*-cyclo­penta­[*b*]thio­phene-3-carboxyl­ate

**DOI:** 10.1107/S1600536812029893

**Published:** 2012-07-07

**Authors:** Jaismary G. B. de Oliveira, Francisco J. B. Mendonça Junior, Maria do Carmo A. de Lima, Carlos A. de Simone, Javier A. Ellena

**Affiliations:** a Laboratório de Síntese e Vetorização de Moléculas Bioativas, Universidade Estadual da Paraíba, 58020-540 João Pessoa, PB, Brazil; b Laboratório de Síntese e Planejamento de Fármacos, Departamento de Antibióticos, Universidade Federal de Pernambuco, 50670-910 Recife, PE, Brazil; c Departamento de Física e Informática, Instituto de Física de São Carlos, Universidade de São Paulo – USP, 13560-970 São Carlos, SP, Brazil

## Abstract

In the title compound, C_17_H_18_N_2_O_2_S_2_, the angle between the mean plane defined by the atoms of the 5,6-dihydro-4*H*-cyclo­penta­[*b*]thio­phene moiety (r.m.s. deviation = 0.19 Å) and the phenyl ring is 72.8°(2). The mol­ecular conformation is stabilized by an intra­molecular N—H⋯O inter­action, which generates an *S*(6) ring motif. In the crystal, pairs of N—H⋯S hydrogen bonds link the mol­ecules to form inversion dimers with an *R*_2_^2^(8) ring motif.

## Related literature

For background to 2-amino­thio­phene derivatives, see: Puterová *et al.* (2010 ▶). For the biological activity of 2-ureido- and 2-thio­ureido-thio­phene-3-carboxyl­ate derivatives, see: Arhin *et al.* (2006 ▶); Saeed *et al.* (2010 ▶). For the synthesis of 2-amino­thio­phenes, see: Gewald *et al.* (1966 ▶). For a related structure, see: Larson & Simonsen (1988 ▶). For hydrogen-bond motifs, see: Bernstein *et al.* (1995 ▶).
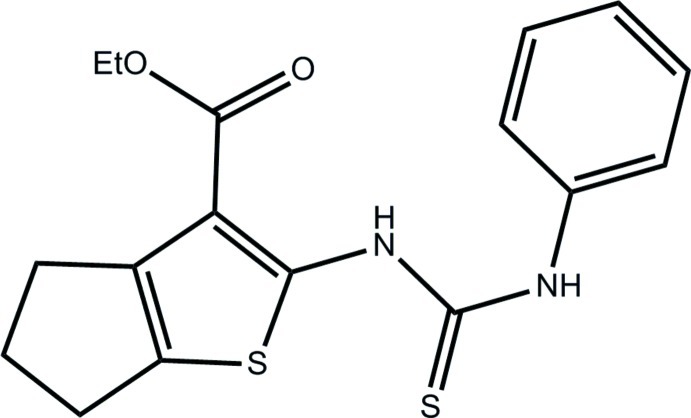


## Experimental

### 

#### Crystal data

C_17_H_18_N_2_O_2_S_2_
*M* *_r_* = 346.45
Triclinic, 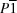

*a* = 5.0755 (2) Å
*b* = 12.5088 (6) Å
*c* = 13.3304 (5) Å
α = 90.562 (3)°
β = 95.711 (3)°
γ = 94.378 (2)°
*V* = 839.61 (6) Å^3^
*Z* = 2
Mo *K*α radiation
μ = 0.33 mm^−1^
*T* = 295 K
0.32 × 0.17 × 0.11 mm

#### Data collection

Nonius KappaCCD diffractometer
9172 measured reflections
3876 independent reflections
2727 reflections with *I* > 2σ(*I*)
*R*_int_ = 0.041

#### Refinement

*R*[*F* ^2^ > 2σ(*F* ^2^)] = 0.045
*wR*(*F* ^2^) = 0.129
*S* = 1.04
3876 reflections
208 parameters
H-atom parameters constrained
Δρ_max_ = 0.28 e Å^−3^
Δρ_min_ = −0.25 e Å^−3^

### 

Data collection: *COLLECT* (Nonius, 1997 ▶); cell refinement: *SCALEPACK* (Otwinowski & Minor, 1997 ▶); data reduction: *DENZO* (Otwinowski & Minor, 1997 ▶) and *SCALEPACK*; program(s) used to solve structure: *SHELXS97* (Sheldrick, 2008 ▶); program(s) used to refine structure: *SHELXL97* (Sheldrick, 2008 ▶); molecular graphics: *ORTEP-3 for Windows* (Farrugia, 1997 ▶); software used to prepare material for publication: *WinGX* (Farrugia, 1999 ▶).

## Supplementary Material

Crystal structure: contains datablock(s) I, global. DOI: 10.1107/S1600536812029893/lr2068sup1.cif

Structure factors: contains datablock(s) I. DOI: 10.1107/S1600536812029893/lr2068Isup2.hkl

Supplementary material file. DOI: 10.1107/S1600536812029893/lr2068Isup3.cml

Additional supplementary materials:  crystallographic information; 3D view; checkCIF report

## Figures and Tables

**Table 1:** Hydrogen-bond geometry (Å, °)

*D*—H⋯*A*	*D*—H	H⋯*A*	*D*⋯*A*	*D*—H⋯*A*
N2—H2⋯S1^i^	0.86	2.61	3.415 (2)	157
N1—H1⋯O1	0.86	2.04	2.719 (2)	136

Symmetry code: (i) 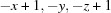.

**Fig. 1.**
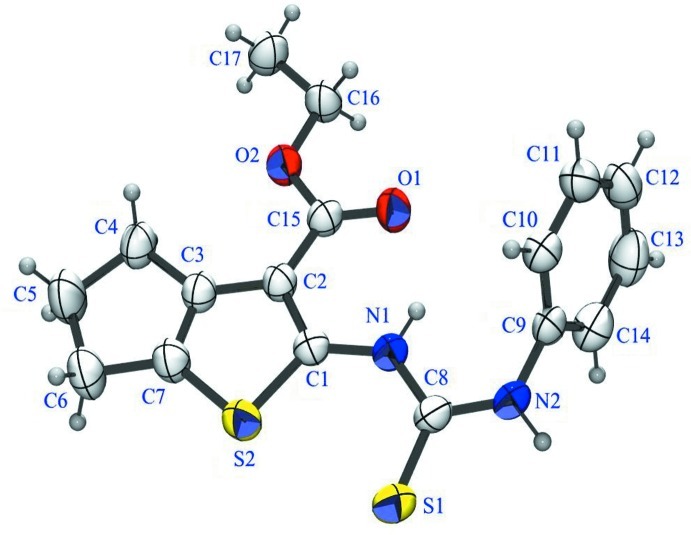
Projection of C17H18N2O2S2, with 50% probability displacement ellipsoids.

**Fig. 2.**
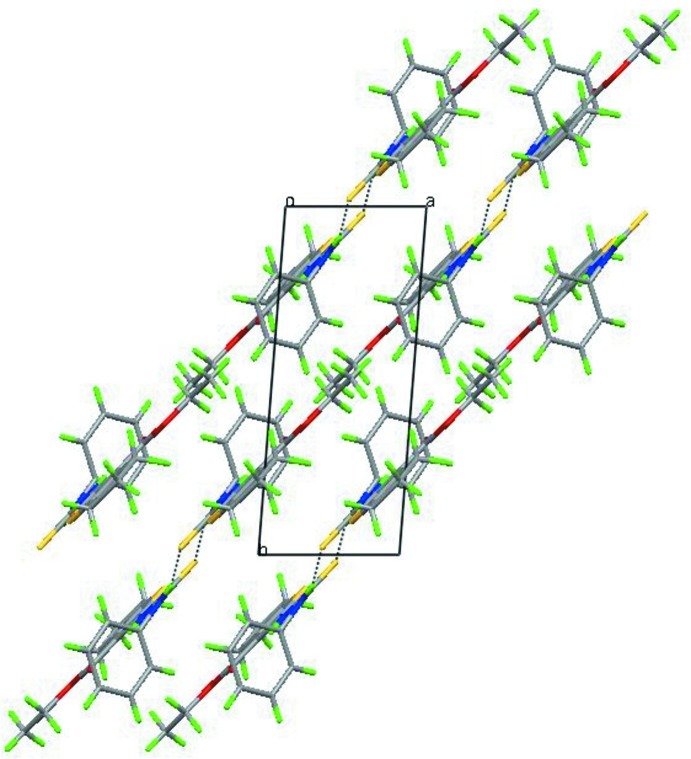
View of the packing along c axis.

## supplementary crystallographic information

### Comment

The various uses and applications of 2-amino thiophene derivatives have been well documented (Puterová *et al.*, 2010). Amongst these appplications, 2-thioureido-thiophene derivatives presents antifungal (Saeed *et al.*, 2010) and antibacterial activities (Arhin *et al.*, 2006). In this work, we report the structure of the title compound prepared by the condensation of 2-amino-5,6-dihydro-4*H*-cyclopenta[*b*]thiophene-3-carbonitrile with phenyl isothiocyanate.

The angle between the least-squares plane defined by the atoms of the 5,6-dihydro-4*H*-cyclopenta[*b*]thiophene moiety (rms deviation=0.19 Å) and the phenyl rings is 72.8°(2). There is an intramolecular N—H···O interaction giving an S(6) ring motif. In the crystal N—H···S hydrogen-bond interactions link the molecules into pairs giving an *R_2_*^2^(8) motif which extends parallel to the plane (120). (Table 2, Fig.2).

### Experimental

Equimolar amounts of 2-amino-5,6-dihydro-4*H*-cyclopenta[*b*]thiophene-3-carbonitrile (4.19 mmol) and phenyl isothiocyanate (4.19 mmol) were heated under reflux for 16 h, in the presence of dry toluene (10 ml), and 5 drops of trietylamine. The solid product formed was collected by filtration, washed with ethyl acetate (3 *x* 10 ml) and crystallized from absolute etanol, affording the title compound as pale yellow crystals (1.07 g, 74%), *M*.p. 185–187 °C. Crystals suitable for single-crystal X-ray diffraction were grown by slow evaporation at room temperature of a solution of the pure title compound in absolute ethanol. NMR ^1^H (400 MHz, CDCl_3_)δ: 1.25 (t, 3H, *J* = 6.4 Hz), 2.28 (d, 2H, *J* = 6.0 Hz), 2.76–2.81 (m, 4H), 4.20 (d, 2H, *J* = 6.0 Hz), 7.24 (s, 1H), 7.39 (d, 2H, *J* = 6.8 Hz), 7.48 (d, 2H, *J* = 7.2 Hz), 11.00 (bs, 1H); 11.58 (bs, 1H).

### Refinement

All H atoms attached to C atoms and N atom were fixed geometrically and treated as riding with C—H = 0.93 Å (aromatic) or 0.97 Å (methylene) and N—H = 0.86 Å with *U*_iso_(H) = 1.2*U*_eq_(C or N).The maximum and minimum residual electron density peaks were located 0.60 and 0.82 Å, from the C2 and S2 atoms respectively.

### Crystal data

**Table:**

C_17_H_18_N_2_O_2_S_2_	*Z* = 2
*M**_r_* = 346.45	*F*(000) = 364
Triclinic, *P*1	*D*_x_ = 1.370 Mg m^−^^3^
Hall symbol: -P 1	Mo *K*α radiation, λ = 0.71073 Å
*a* = 5.0755 (2) Å	Cell parameters from 5829 reflections
*b* = 12.5088 (6) Å	θ = 2.6–27.5°
*c* = 13.3304 (5) Å	µ = 0.33 mm^−^^1^
α = 90.562 (3)°	*T* = 295 K
β = 95.711 (3)°	Prism, yellow
γ = 94.378 (2)°	0.32 × 0.17 × 0.11 mm
*V* = 839.61 (6) Å^3^	

### Data collection

**Table:**

Nonius KappaCCD diffractometer	2727 reflections with *I* > 2σ(*I*)
Radiation source: Enraf Nonius FR590	*R*_int_ = 0.041
Horizonally mounted graphite crystal monochromator	θ_max_ = 27.5°, θ_min_ = 3.1°
Detector resolution: 9 pixels mm^-1^	*h* = −5→6
CCD rotation images,thick slices scans	*k* = −16→16
9172 measured reflections	*l* = −17→17
3876 independent reflections	

### Refinement

**Table:**

Refinement on *F*^2^	Primary atom site location: structure-invariant direct methods
Least-squares matrix: full	Secondary atom site location: difference Fourier map
*R*[*F*^2^ > 2σ(*F*^2^)] = 0.045	Hydrogen site location: inferred from neighbouring sites
*wR*(*F*^2^) = 0.129	H-atom parameters constrained
*S* = 1.04	*w* = 1/[σ^2^(*F*_o_^2^) + (0.0607*P*)^2^ + 0.2145*P*] where *P* = (*F*_o_^2^ + 2*F*_c_^2^)/3
3876 reflections	(Δ/σ)_max_ = 0.001
208 parameters	Δρ_max_ = 0.28 e Å^−^^3^
0 restraints	Δρ_min_ = −0.25 e Å^−^^3^

### Special details

**Table:**

Geometry. All e.s.d.'s (except the e.s.d. in the dihedral angle between two l.s. planes) are estimated using the full covariance matrix. The cell e.s.d.'s are taken into account individually in the estimation of e.s.d.'s in distances, angles and torsion angles; correlations between e.s.d.'s in cell parameters are only used when they are defined by crystal symmetry. An approximate (isotropic) treatment of cell e.s.d.'s is used for estimating e.s.d.'s involving l.s. planes.
Refinement. Refinement of *F*^2^ against ALL reflections. The weighted *R*-factor *wR* and goodness of fit *S* are based on *F*^2^, conventional *R*-factors *R* are based on *F*, with *F* set to zero for negative *F*^2^. The threshold expression of *F*^2^ > σ(*F*^2^) is used only for calculating *R*-factors(gt) *etc*. and is not relevant to the choice of reflections for refinement. *R*-factors based on *F*^2^ are statistically about twice as large as those based on *F*, and *R*- factors based on ALL data will be even larger.

### Fractional atomic coordinates and isotropic or equivalent isotropic displacement parameters (Å^2^)

**Table:**

	*x*	*y*	*z*	*U*_iso_*/*U*_eq_	
S1	0.55314 (12)	0.02072 (4)	0.67471 (4)	0.05884 (19)	
S2	0.35795 (11)	0.09492 (4)	0.87171 (4)	0.05360 (17)	
O2	−0.2636 (3)	0.37540 (12)	0.83065 (10)	0.0528 (4)	
O1	−0.1299 (3)	0.33086 (13)	0.68141 (10)	0.0607 (4)	
N1	0.2196 (3)	0.17647 (13)	0.68280 (11)	0.0469 (4)	
H1	0.1318	0.2229	0.6494	0.056*	
N2	0.3310 (4)	0.13297 (14)	0.52705 (13)	0.0582 (5)	
H2	0.3999	0.0896	0.4885	0.070*	
C1	0.1968 (4)	0.17638 (15)	0.78532 (14)	0.0423 (4)	
C9	0.1970 (4)	0.21777 (16)	0.47921 (14)	0.0487 (5)	
C10	0.3005 (4)	0.32279 (17)	0.49491 (15)	0.0536 (5)	
H10	0.4551	0.3384	0.5377	0.064*	
C2	0.0363 (4)	0.24425 (15)	0.83011 (13)	0.0425 (4)	
C4	−0.0737 (4)	0.2761 (2)	1.02319 (15)	0.0566 (5)	
H4A	−0.2659	0.2660	1.0134	0.068*	
H4B	−0.0208	0.3521	1.0313	0.068*	
C8	0.3604 (4)	0.11405 (15)	0.62679 (15)	0.0466 (4)	
C6	0.2245 (5)	0.1327 (2)	1.07959 (16)	0.0670 (6)	
H6A	0.4038	0.1475	1.1117	0.080*	
H6B	0.1620	0.0597	1.0934	0.080*	
C11	0.1727 (5)	0.40396 (19)	0.44669 (17)	0.0635 (6)	
H11	0.2408	0.4748	0.4573	0.076*	
C15	−0.1225 (4)	0.31881 (16)	0.77266 (14)	0.0459 (4)	
C3	0.0489 (4)	0.22821 (16)	0.93666 (14)	0.0468 (4)	
C16	−0.4291 (5)	0.45183 (19)	0.77988 (16)	0.0573 (5)	
H16A	−0.5613	0.4152	0.7313	0.069*	
H16B	−0.3218	0.5036	0.7446	0.069*	
C7	0.2103 (4)	0.15241 (18)	0.96808 (15)	0.0533 (5)	
C13	−0.1550 (5)	0.2766 (3)	0.36669 (18)	0.0747 (7)	
H13	−0.3081	0.2614	0.3229	0.090*	
C14	−0.0298 (5)	0.1938 (2)	0.41490 (17)	0.0635 (6)	
H14	−0.0979	0.1230	0.4040	0.076*	
C12	−0.0536 (5)	0.3809 (2)	0.38337 (18)	0.0704 (7)	
H12	−0.1395	0.4362	0.3514	0.084*	
C17	−0.5609 (5)	0.5069 (2)	0.85914 (18)	0.0677 (6)	
H17A	−0.6727	0.5584	0.8282	0.102*	
H17B	−0.4280	0.5428	0.9067	0.102*	
H17C	−0.6665	0.4548	0.8934	0.102*	
C5	0.0370 (7)	0.2135 (3)	1.11409 (18)	0.0839 (8)	
H5A	−0.1079	0.1758	1.1448	0.101*	
H5B	0.1328	0.2628	1.1640	0.101*	

### Atomic displacement parameters (Å^2^)

**Table:**

	*U*^11^	*U*^22^	*U*^33^	*U*^12^	*U*^13^	*U*^23^
S1	0.0707 (4)	0.0530 (3)	0.0557 (3)	0.0257 (3)	0.0055 (3)	−0.0062 (2)
S2	0.0602 (3)	0.0550 (3)	0.0490 (3)	0.0214 (2)	0.0087 (2)	0.0070 (2)
O2	0.0585 (8)	0.0617 (9)	0.0428 (7)	0.0272 (7)	0.0098 (6)	0.0028 (6)
O1	0.0755 (10)	0.0716 (10)	0.0404 (7)	0.0340 (8)	0.0104 (7)	0.0052 (7)
N1	0.0571 (10)	0.0472 (9)	0.0395 (8)	0.0183 (7)	0.0090 (7)	−0.0002 (7)
N2	0.0839 (13)	0.0522 (10)	0.0443 (9)	0.0294 (9)	0.0165 (9)	−0.0042 (7)
C1	0.0445 (10)	0.0414 (9)	0.0419 (9)	0.0075 (8)	0.0061 (8)	0.0001 (7)
C9	0.0612 (12)	0.0511 (11)	0.0376 (9)	0.0169 (9)	0.0151 (9)	−0.0015 (8)
C10	0.0628 (12)	0.0535 (12)	0.0462 (11)	0.0113 (10)	0.0092 (9)	−0.0018 (9)
C2	0.0430 (9)	0.0469 (10)	0.0388 (9)	0.0080 (8)	0.0062 (7)	0.0016 (7)
C4	0.0593 (12)	0.0712 (14)	0.0419 (10)	0.0127 (11)	0.0123 (9)	−0.0014 (9)
C8	0.0542 (11)	0.0401 (10)	0.0469 (10)	0.0080 (8)	0.0100 (9)	−0.0062 (8)
C6	0.0708 (14)	0.0866 (17)	0.0461 (11)	0.0164 (13)	0.0095 (11)	0.0156 (11)
C11	0.0840 (16)	0.0557 (13)	0.0551 (12)	0.0173 (12)	0.0192 (12)	0.0064 (10)
C15	0.0472 (10)	0.0495 (11)	0.0431 (10)	0.0119 (8)	0.0094 (8)	−0.0015 (8)
C3	0.0480 (10)	0.0530 (11)	0.0401 (10)	0.0066 (9)	0.0062 (8)	0.0001 (8)
C16	0.0640 (13)	0.0641 (13)	0.0481 (11)	0.0295 (11)	0.0071 (10)	0.0053 (9)
C7	0.0558 (12)	0.0611 (13)	0.0453 (10)	0.0139 (10)	0.0089 (9)	0.0065 (9)
C13	0.0649 (15)	0.108 (2)	0.0526 (13)	0.0221 (15)	0.0002 (11)	−0.0051 (13)
C14	0.0652 (14)	0.0703 (15)	0.0556 (13)	0.0062 (12)	0.0090 (11)	−0.0085 (11)
C12	0.0844 (17)	0.0814 (18)	0.0520 (13)	0.0384 (14)	0.0141 (12)	0.0134 (12)
C17	0.0762 (15)	0.0731 (15)	0.0578 (13)	0.0338 (13)	0.0067 (11)	−0.0080 (11)
C5	0.109 (2)	0.103 (2)	0.0471 (13)	0.0402 (18)	0.0182 (14)	0.0121 (13)

### Geometric parameters (Å, º)

**Table:**

S1—C8	1.671 (2)	C4—H4B	0.9700
S2—C7	1.728 (2)	C6—C7	1.505 (3)
S2—C1	1.7310 (19)	C6—C5	1.537 (4)
O2—C15	1.337 (2)	C6—H6A	0.9700
O2—C16	1.449 (2)	C6—H6B	0.9700
O1—C15	1.224 (2)	C11—C12	1.366 (4)
N1—C8	1.363 (2)	C11—H11	0.9300
N1—C1	1.383 (2)	C3—C7	1.343 (3)
N1—H1	0.8600	C16—C17	1.495 (3)
N2—C8	1.348 (3)	C16—H16A	0.9700
N2—C9	1.425 (3)	C16—H16B	0.9700
N2—H2	0.8600	C13—C12	1.374 (4)
C1—C2	1.391 (3)	C13—C14	1.386 (4)
C9—C14	1.377 (3)	C13—H13	0.9300
C9—C10	1.383 (3)	C14—H14	0.9300
C10—C11	1.377 (3)	C12—H12	0.9300
C10—H10	0.9300	C17—H17A	0.9600
C2—C3	1.432 (3)	C17—H17B	0.9600
C2—C15	1.453 (3)	C17—H17C	0.9600
C4—C3	1.504 (3)	C5—H5A	0.9700
C4—C5	1.532 (3)	C5—H5B	0.9700
C4—H4A	0.9700		
			
C7—S2—C1	90.32 (9)	C10—C11—H11	119.9
C15—O2—C16	116.57 (15)	O1—C15—O2	122.23 (17)
C8—N1—C1	129.72 (17)	O1—C15—C2	125.21 (17)
C8—N1—H1	115.1	O2—C15—C2	112.56 (16)
C1—N1—H1	115.1	C7—C3—C2	112.96 (18)
C8—N2—C9	126.40 (16)	C7—C3—C4	111.40 (18)
C8—N2—H2	116.8	C2—C3—C4	135.63 (18)
C9—N2—H2	116.8	O2—C16—C17	107.08 (17)
N1—C1—C2	122.14 (17)	O2—C16—H16A	110.3
N1—C1—S2	125.40 (14)	C17—C16—H16A	110.3
C2—C1—S2	112.45 (14)	O2—C16—H16B	110.3
C14—C9—C10	120.6 (2)	C17—C16—H16B	110.3
C14—C9—N2	119.4 (2)	H16A—C16—H16B	108.6
C10—C9—N2	119.87 (19)	C3—C7—C6	114.13 (19)
C11—C10—C9	119.5 (2)	C3—C7—S2	113.40 (16)
C11—C10—H10	120.3	C6—C7—S2	132.46 (17)
C9—C10—H10	120.3	C12—C13—C14	120.2 (2)
C1—C2—C3	110.87 (17)	C12—C13—H13	119.9
C1—C2—C15	122.59 (16)	C14—C13—H13	119.9
C3—C2—C15	126.54 (17)	C9—C14—C13	119.1 (2)
C3—C4—C5	103.25 (18)	C9—C14—H14	120.5
C3—C4—H4A	111.1	C13—C14—H14	120.5
C5—C4—H4A	111.1	C11—C12—C13	120.4 (2)
C3—C4—H4B	111.1	C11—C12—H12	119.8
C5—C4—H4B	111.1	C13—C12—H12	119.8
H4A—C4—H4B	109.1	C16—C17—H17A	109.5
N2—C8—N1	114.24 (17)	C16—C17—H17B	109.5
N2—C8—S1	121.58 (14)	H17A—C17—H17B	109.5
N1—C8—S1	124.18 (15)	C16—C17—H17C	109.5
C7—C6—C5	101.64 (19)	H17A—C17—H17C	109.5
C7—C6—H6A	111.4	H17B—C17—H17C	109.5
C5—C6—H6A	111.4	C4—C5—C6	109.55 (19)
C7—C6—H6B	111.4	C4—C5—H5A	109.8
C5—C6—H6B	111.4	C6—C5—H5A	109.8
H6A—C6—H6B	109.3	C4—C5—H5B	109.8
C12—C11—C10	120.3 (2)	C6—C5—H5B	109.8
C12—C11—H11	119.9	H5A—C5—H5B	108.2

### Hydrogen-bond geometry (Å, º)

**Table:**

*D*—H···*A*	*D*—H	H···*A*	*D*···*A*	*D*—H···*A*
N2—H2···S1^i^	0.86	2.61	3.415 (2)	157
N1—H1···O1	0.86	2.04	2.719 (2)	136

Symmetry code: (i) −*x*+1, −*y*, −*z*+1.
